# Metabolomic Impact of Maternal Barium Exposure on Miscarriage Risk: Identification of Metabolic Biomarkers and Construction of a Risk Prediction Model

**DOI:** 10.3390/toxics13121066

**Published:** 2025-12-10

**Authors:** Xiaoyu Zhao, Ziwei Guo, Shuangshuang Zhao, Danyang Wan, Jie Xu, Yifan Xu, Yujie Liu, Haoyi Xu, Ziyang Wang, Qing Xu

**Affiliations:** 1 Women’s Hospital of Nanjing Medical University, Nanjing Women and Children’s Healthcare Hospital, Nanjing 210004, China; 2 State Key Laboratory of Reproductive Medicine and Offspring Health, School of Public Health, Nanjing Medical University, Nanjing 211166, China; 3 Key Laboratory of Modern Toxicology of Ministry of Education, School of Public Health, Nanjing Medical University, Nanjing 211166, China

**Keywords:** prenatal barium exposure, miscarriage, metabolomics, mediation

## Abstract

This study investigated the relationship between maternal barium (Ba) exposure and the risk of miscarriage using metabolomics and machine learning. Analyses were performed on samples from 183 pregnant women from Nanjing: the concentration of Ba in whole blood was measured using inductively coupled plasma mass spectrometry (ICP-MS), and untargeted metabolomics was performed on decidual tissue using high-resolution accurate mass spectrometry (UHPLC-QExactive HF-X). A metabolome-wide association study (MWAS) and mediation interaction effect analysis (MITM) identified metabolites and pathways linked to Ba exposure and miscarriage risk. Among 523 detected metabolites, 19 metabolites and 5 pathways were significantly associated with both Ba exposure and miscarriage, particularly glycerophospholipid metabolism. The effect of Ba exposure on miscarriage risk was mediated by five metabolites, with cuminaldehyde showing the highest share of the total mediating effect (54.74%). These metabolites, including N-acetyl-L-methionine, 4-hydroxynonenal, DG(18:0/18:3(9Z,12Z,15Z)/0:0), 10-formyldihydrofolate, and eicosadienoic acid, were used as biomarkers in a predictive model. The XGBoost model achieved an optimal AUC of 0.90 (95%CI: 0.83–0.96). This study suggests that maternal Ba exposure increases miscarriage risk, potentially through disruptions in amino acid metabolism, oxidative stress, and lipid peroxidation, and highlights the potential of metabolite biomarkers for predicting adverse birth outcomes.

## 1. Introduction

Miscarriage, also called early spontaneous abortion (ESA), is defined as the spontaneous termination of pregnancy before 20 weeks of gestation or when the fetal weight is less than 500 g. Miscarriage is a major issue in global reproductive health [1]. In recent years, the incidence of miscarriage has steadily increased, becoming one of the leading causes of adverse pregnancy outcomes. The causes of miscarriage are complex; they include genetic and chromosomal abnormalities, infections, thrombotic diseases, anatomical defects, endocrine disorders, and immune dysfunction [2,3]. However, approximately 50% of miscarriage cases remain unexplained. Increasing evidence suggests that environmental factors may play a crucial role in the risk of miscarriage, with environmental pollutants, particularly heavy metals, being a focus of research due to their long-term accumulation and significant toxic effects, which can result from both maternal exposure and transgenerational pathways [4,5,6].

As a heavy metal element prevalent in the environment, barium (Ba) is commonly found in the Earth’s crust and groundwater. Anthropogenic sources of Ba include plastic manufacturing and processing, pesticide spraying, diesel engine emissions, and pharmaceutical use [7]. Ba can enter the bloodstream through ingestion, inhalation, or dermal contact, and it may accumulate in the placenta. The World Health Organization (WHO) has established a safe reference value for barium in drinking water [7,8]. Prolonged Ba exposure might threaten human health. In recent studies, maternal Ba exposure was closely associated with adverse pregnancy outcomes, such as miscarriage [9,10]. Our previous case–control study established a significant positive correlation between maternal blood Ba levels and the risk of early spontaneous abortion (ESA) [11]. Previous studies showed that once Ba entered the maternal body, it dispersed in the blood in its ionic form and could cross the maternal–fetal interface, thereby affecting embryonic development [12]. Research has shown that Ba causes oxidative-stress-induced damage not only to placental tissue [13] but also to mitochondrial DNA, leading to dysfunctional cellular energy metabolism [14]. The study further indicated that Ba may impair telomeres and reduce their repair efficiency, ultimately leading to adverse pregnancy outcomes such as miscarriage [15,16]. However, the specific mechanisms by which barium exposure induces early pregnancy loss remain unclear and warrant further investigation.

Metabolomics, a comprehensive analysis of small molecules in biological samples, offers a powerful tool for elucidating the mechanisms underlying environmental pollutants’ effects on adverse birth outcomes [17,18]. In recent years, an increasing number of studies have recognized the significant role of metabolic abnormalities in miscarriage, identifying biomarkers associated with glucose, lipid, and amino acid metabolism, as well as redox balance [19]. However, previous studies have largely focused solely on either environmental exposure or metabolic analyses, without integrating both aspects to provide a comprehensive understanding. Tong [20] reported that Ba exposure in utero and in early childhood was positively correlated with glucose and triglyceride levels in preschool children, while it was negatively correlated with high-density lipoprotein cholesterol. However, the mechanisms by which barium exposure affects pregnancy maintenance, particularly through endogenous biochemical mechanisms and metabolic disruptions, remain inadequately understood. Specifically, the molecular pathways, biomarkers, and mediating roles between barium exposure and spontaneous abortion have not been fully elucidated.

Building on our previous study, in which we found a significant positive correlation between maternal blood Ba levels and the risk of ESA [11], the current analysis further explores the relationship between barium and metabolic changes in pregnant women to address the abovementioned research gap. We combined measurements of Ba in maternal whole blood with untargeted metabolomic profiling of decidual tissue, aiming to uncover the underlying mechanisms of this association and provide new insights. We performed a metabolome-wide association study (MWAS) and mediation analysis to identify critical metabolites and pathways linked to both Ba exposure and miscarriage and to quantify their potential mediating roles. Furthermore, we developed and validated a miscarriage prediction model by integrating Ba exposure data with metabolite biomarkers using machine learning algorithms. This study aims to delineate the metabolic mechanisms underlying Ba-induced miscarriage and to provide novel biomarkers for risk assessment and etiological clarification.

This study aimed to reveal how Ba exposure induces miscarriage and identify key biomarkers and molecular mechanisms. Through systematic metabolomic analysis, we sought not only to uncover the metabolic disturbances caused by Ba exposure but also to offer new insights and evidence for identifying biomarkers and predicting the risk of miscarriage.

## 2. Materials and Methods

### 2.1. Study Design and Population

This cross-sectional study was conducted at the Women’s Hospital of Nanjing Medical University (Nanjing Women and Children’s Healthcare Hospital) between September 2020 and February 2021. The study enrolled pregnant women who were in good physical health, with no acute medical conditions (such as severe cardiovascular, hepatic, or renal diseases, reproductive tract infections, or immune-related disorders), and no history of illicit drug use. The inclusion criteria were strictly limited to pregnant women aged 18 years and older with a gestational age of ≤12 weeks. A total of 528 participants were enrolled in the baseline survey. They completed a comprehensive questionnaire, medical record review, physical examination, and auxiliary tests, including serum and urine β-human chorionic gonadotropin (β-hCG) measurements and pelvic ultrasound examination.

The study participants were divided into two groups: the miscarriage group (cases) and the control group (healthy pregnant women). The case group consisted of individuals diagnosed with spontaneous abortion, based on the criteria from the American College of Obstetricians and Gynecologists and supplementary examination results [21]. The diagnostic criteria included the absence of or decline in β-hCG based on dynamic monitoring values, as well as ultrasound results meeting at least one of the following standards: crown–rump length (CRL) ≥ 7 mm with no heartbeat, mean sac diameter (MSD) ≥ 25 mm with no embryo, or no fetal heartbeat within the designated time window after the detection of the gestational sac (no yolk sac ≥ 2 weeks, with yolk sac ≥ 11 days). The control group consisted of healthy pregnant women who were ≤12 weeks pregnant, with an ultrasound showing a heartbeat, and who chose to undergo an induced abortion for non-medical reasons. Gestational age was calculated from the first day of the last menstrual period. After excluding participants with chromosomal abnormalities, underlying diseases (uterine diseases, thyroid disorders, etc.; *n* = 68), missing whole-blood samples (*n* = 239), or missing covariate information (*n* = 26) and those without decidual tissue samples (*n* = 12), a total of 183 pregnant women who had signed informed consent forms were included in the analysis. Whole-blood samples for trace element concentration measurements were collected at the time of pregnancy diagnosis, while decidual tissue for metabolomic analysis was obtained during a clinical curettage procedure performed subsequent to the diagnosis. The study was approved by the Medical Ethics Committee of Nanjing Maternity and Child Health Hospital (2020-KY-054).

### 2.2. Blood Collection and Elemental Detection

The concentrations of trace elements in maternal whole blood were determined using an inductively coupled plasma mass spectrometry (ICP-MS) method, which was previously established and validated in our laboratory (Thermo Scientific, Bremen, Germany) [11]. Blood samples were stored at −80 °C and transported to the laboratory, where 100 μL of each sample was transferred to centrifuge tubes pre-treated with acid. A mixture of 1% nitric acid and 0.1% Triton X-100 containing internal standards was added to the samples, which were then digested overnight at 4 °C. The final volume was adjusted to 5.0 mL for analysis. Sample analysis was performed using optimized ICP-MS parameters. Each batch of analyses included calibration with a standard (Seronorm^TM^ Trace Elements Whole Blood L-1; SERO AS, Billingstad, Norway), process blanks, pooled quality control (QC) samples, and a negative control (deionized water). Samples were randomized and analyzed blindly to minimize bias.

All elements met acceptance criteria, with recovery rates between 90% and 110%, and relative standard deviations (RSDs) of <12%. In total, 20 trace elements were analyzed in this study. The limit of detection (LOD) for each element was calculated as three times the standard deviation of blank values, and concentrations below the LOD were replaced with half of the LOD. Based on conventional practices outlined in the literature [22], only elements with a detection rate >80% were included in subsequent epidemiological analyses to ensure statistical reliability. A total of 11 elements were retained for further analysis: arsenic (As), barium (Ba), caesium (Cs), copper (Cu), iron (Fe), manganese (Mn), lead (Pb), rubidium (Rb), strontium (Sr), vanadium (V), and zinc (Zn).

Based on previous findings in the same cohort [11], Ba levels in maternal blood were established as the core exposure indicator for this metabolomics study and were used for subsequent association analyses.

### 2.3. Tissue Collection and Metabolomic Analysis

In this study, decidual tissue was selected for metabolomic analysis due to its crucial role as a key structure at the maternal–fetal interface, directly involved in regulating critical processes such as embryo implantation, immune tolerance, and angiogenesis [23]. The metabolic state of the decidua is likely to be more sensitive in reflecting early pregnancy changes and can provide insights into local metabolic alterations associated with maternal-fetal interactions [24]. The collected decidual tissue samples were immediately snap-frozen in liquid nitrogen and then stored at −80 °C in a freezer at the Women’s Hospital of Nanjing Medical University, Nanjing Women and Children’s Healthcare Hospital, for subsequent metabolomic analysis. In this study, non-targeted metabolomic analysis of the decidual tissue samples was performed using an ultra-high-performance liquid chromatography-equipped quadrupole-exactive high-resolution mass spectrometry (UHPLC-QExactiveHF-X, Thermo Fisher Scientific) system. For sample preparation, 50 mg of decidual tissue was accurately weighed and extracted with a methanol–water (4:1, *v*:*v*) extraction solvent containing 0.02 mg/mL L-2-chlorophenylalanine as an internal standard. After freezing the tissue at −10 °C and grinding it, the sample was subjected to low-temperature ultrasound at 5 °C. The proteins were precipitated, and the supernatant was collected by centrifugation at 13,000× *g*. For quality control, a QC sample, prepared by mixing equal volumes of all samples, was inserted every 5–15 samples to monitor batch reproducibility.

Chromatographic separation was performed using an HSST3 column (100 mm × 2.1 mm i.d., 1.8 μm) at a flow rate of 0.40 mL/min and column temperature of 40 °C. Mobile phase A consisted of 95% water + 5% acetonitrile (with 0.1% formic acid), and mobile phase B was 47.5% acetonitrile + 47.5% isopropanol + 5% water (with 0.1% formic acid). A 15 min gradient elution was applied (key steps: 0.0–1.0 min, 5% B; 1.0–9.0 min, linearly increasing to 95% B; 9.0–12.0 min, maintaining 95% B; then rapidly returning to initial conditions). Mass spectrometry was performed in data-dependent acquisition (DDA) mode with positive- and negative-ion scans. The resolution was 60,000 for MS1 and 7500 for MS2, with collision energies of 20, 40, and 60 V in cycles. The ion spray voltage was set at ±3500 V, and the scan range was 70–1050 *m*/*z*. Raw data were preprocessed and normalized, and missing values >40% were excluded using ProteoWizard 3.x and XCMS 3.x.

Metabolites were identified based on molecular mass errors < 10 ppm, retention time, and MS/MS fragmentation patterns, referencing several online databases, including METLIN, Kyoto Encyclopedia of Genes and Genomes (KEGG), Human Metabolome Database (HMDB), and LIPID MAPS, complemented by theoretical fragment identification.

### 2.4. Covariate Assessment

Through structured face-to-face interviews, we systematically collected data on sociodemographic characteristics, obstetric history, and lifestyle habits of the participants. These data included maternal age (years), pre-pregnancy body mass index (BMI [kg/m^2^]), gestational age, prior pregnancies and prior miscarriages, prior live births, education level, occupation, personal income, and smoking and alcohol consumption during pregnancy. Additionally, all information related to pregnancy and health conditions was obtained and supplemented through electronic medical records.

### 2.5. Statistical Analysis

Differences between groups for categorical and continuous variables were compared using Chi-square tests and *t*-tests, respectively. As demonstrated in our previous publication [11], logistic regression models, WQS, and BKMR methods revealed a significant positive association between whole-blood Ba exposure and miscarriage. Based on this finding, the present study focused on exploring the association between maternal whole-blood Ba levels and the metabolic profiles of decidual tissue to reveal potential biological mechanisms.

Before conducting subsequent analyses, exploratory analyses of the metabolomics data were performed using principal component analysis (PCA) and orthogonal partial least squares discriminant analysis (OPLS-DA). An MWAS was employed to investigate the associations between decidual tissue metabolomics, the concentration of Ba, and miscarriage. For the untargeted MWAS, generalized linear models (GLMs) were applied to examine the association between the Ba concentration and the metabolic feature intensities associated with miscarriage. Metabolite levels were log10-transformed for data normalization, as they exhibited right-skewed distributions. All association models were adjusted for the same set of covariates, including maternal age, gestational age, pre-pregnancy BMI, education levels, and prior live births.

Subsequently, metabolic pathways significantly associated with Ba exposure and miscarriage were identified using the MetaboAnalyst 6.0 platform (https://www.metaboanalyst.ca/) and the KEGG database (for Homo sapiens) [25,26,27]. In the pathway analysis, after mapping metabolites to HMDB/KEGG IDs, over-representation analysis was performed using a global test. The MITM approach, previously used in environmental pollutant and reproductive health studies to explore early biological effects and identify potential intermediate biomarkers, was employed in this study. The MITM approach identified overlapping metabolites and pathways that were significantly associated with both the Ba concentration and miscarriage and was used to assess their mediating effects on the relationship between maternal Ba exposure and miscarriage [28]. However, due to the limited sample size, unadjusted *p* values (*p* < 0.05) were used in this exploratory MITM analysis.

Stratified sampling was applied to divide the data into training and testing sets in a 6:4 ratio. Various algorithms, including logistic regression (LR), random forest (RF), eXtreme Gradient Boosting (XGBoost), and Light Gradient Boosting Machine (LightGBM), were used to construct prediction models with miscarriage as the dependent variable and the concentration of Ba in whole blood and differential metabolites as independent variables. To identify core biomarkers, two complementary feature selection strategies were combined: Least Absolute Shrinkage and Selection Operator (LASSO) logistic regression, using five-fold cross-validation to determine the optimal λ value and retain non-zero coefficient variables, and RF, which initially screened the top 10 important metabolites based on the Gini index decrease. Subsequently, correlation analysis among metabolites was performed to remove collinear variables (*r* > 0.8), and the intersection of LASSO and RF results was used to identify the final 5 core metabolites as the feature set. Model performance was evaluated using five-fold cross-validation, and the model’s discriminatory power was quantified through Receiver Operating Characteristic (ROC) curve analysis and the area under the curve (AUC). The performance of different models was compared using accuracy, recall, precision, and F1 score, and the best-performing model was selected as the final model. In terms of result presentation, the scientific rigor of the model was assessed using calibration curves, learning curves, and residual plots; prediction ability was measured using Precision–Recall (PR) curves and their AUPR metrics; and clinical utility was determined by decision curve analysis (DCA). Finally, SHAP methods were used to perform interpretability analysis of the best model to further explore the mechanisms of key biomarkers.

All statistical analyses were performed in R (v4.4.1), with a significance level set at *p* < 0.05 for all statistical tests. Mediation analysis was performed using the “mediation” R package.

## 3. Results

### 3.1. Inclusion of Study Subjects and Baseline Characteristics

**Figure 1:**
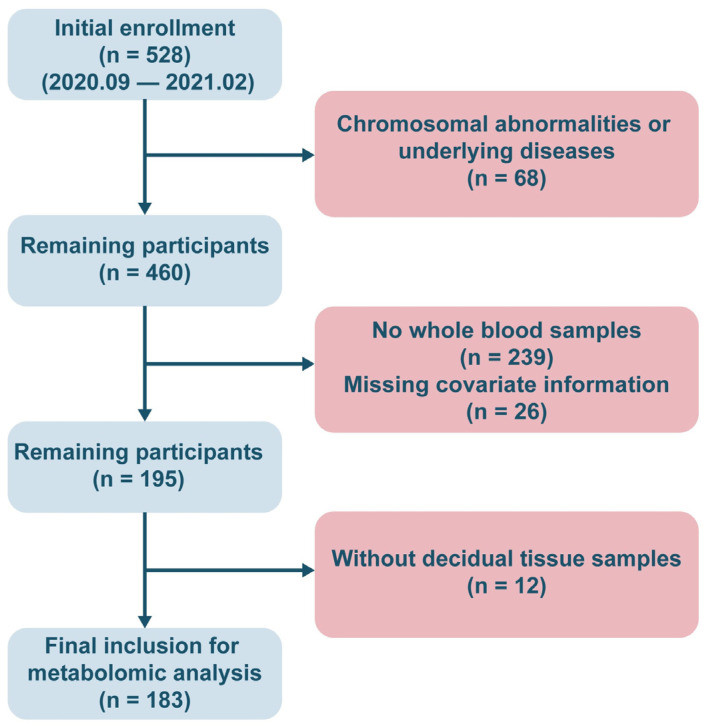
The flowchart of the inclusion of study population.

This study followed a rigorous inclusion and exclusion process, ultimately enrolling 183 pregnant women who met the criteria for analysis (miscarriage group, *n* = 82; control group, *n* = 101). A flowchart of the sample selection process is shown in Figure 1. The basic sociodemographic characteristics, clinical information, and distribution of potential confounders for the study subjects are summarized in Table 1. No statistically significant differences in age, pre-pregnancy BMI, or other covariates were observed between the miscarriage and control groups (*p* > 0.05). The miscarriage group contained a significantly higher proportion of women with a history of ≥1 prior miscarriage (*p* < 0.05), whereas the control group was predominantly composed of women reporting ≥1 prior live birth (*p* < 0.001).

**Figure 2:**
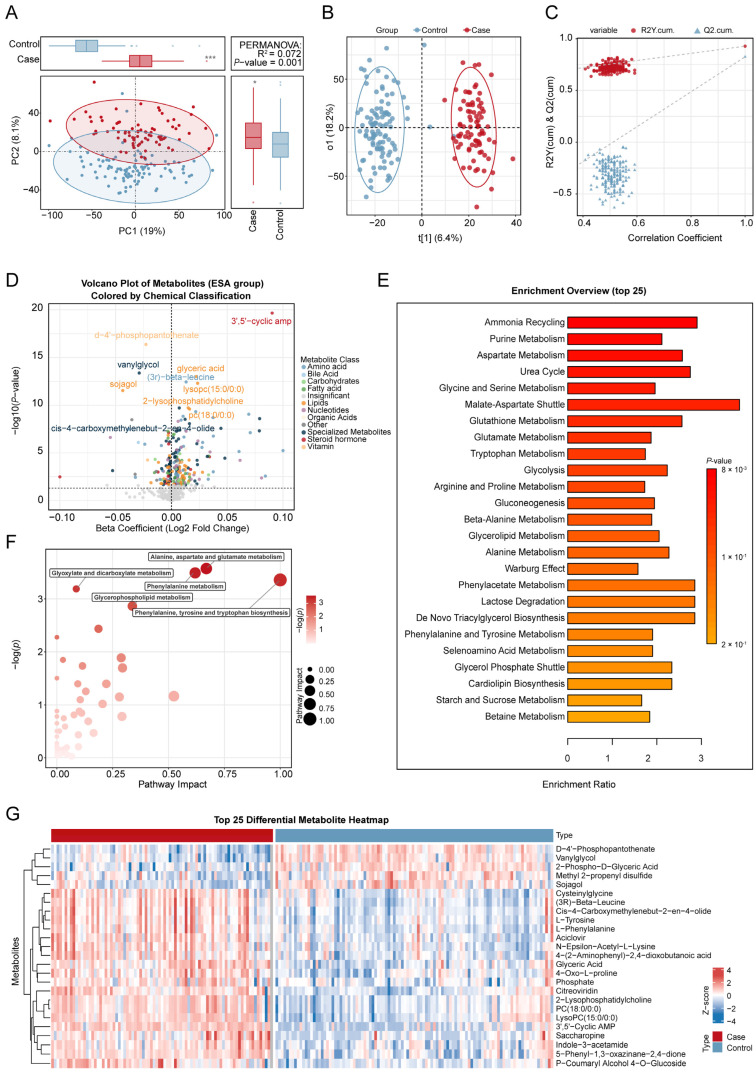
Comprehensive metabolomic analysis of the risk of miscarriage. (**A**). Principal component analysis (PCA) of untargeted metabolomics data from decidual tissue comparing miscarriage and control groups. (**B**). The score plot of the orthogonal partial least squares discriminant analysis (OPLS-DA) model. (**C**). The permutation test plot for metabolite differences between the miscarriage and control groups. (**D**). The volcano plot of the association between metabolites and the risk of miscarriage, analyzed by linear regression models. The *x*-axis shows the estimated coefficient of the association between metabolites in trophoblast tissue and the risk of miscarriage, and the *y*-axis shows its −log10 value (*p* value). The horizontal dotted line represents the significance threshold (*p* value = 0.05). (**E**). Overview of the top 25 enriched metabolic pathways associated with the risk of miscarriage. (**F**). Pathway enrichment analysis of miscarriage-related metabolites. (**G**). Top 25 differential metabolites between the miscarriage and control groups. Covariates include maternal age, gestational age, pre-pregnancy BMI, education levels, and prior live births. * *p* < 0.05，*** *p* < 0.001.

Before conducting the association analysis, we first assessed the quality of the decidual tissue metabolomics data and the separation between groups. A scatter plot of PCA results (Figure 2A) showed an initial trend of separation between the miscarriage and control groups in the principal component space, suggesting potential differences in metabolic profiles between the groups. To maximize the inter-group variation, we further applied OPLS-DA. The OPLS-DA model successfully achieved significant separation between the two groups (Figure 2B), with good model fit (R^2^Y = 0.93) and predictive ability (Q^2^ = 0.83). The permutation test results for the model (Figure 2C) indicated that it was robust and not overfitted (*p* < 0.05).

### 3.2. Maternal Blood Ba Exposure and Its Association with Miscarriage: A Metabolomics Study (MWAS)

The median concentration of Ba in maternal whole blood was significantly higher in the miscarriage group (6.16 ng/mL) than in the control group (4.33 ng/mL; *p* < 0.05). Ba levels were significantly elevated in the miscarriage group compared to controls and also exhibited greater variance. The association between the concentration of maternal blood Ba and the risk of spontaneous abortion has been previously established by our team [11]. In the current study, we further investigated the metabolomic impact of Ba exposure in decidual tissue. Of the 523 detected metabolites (Table S1), 49 were significantly correlated with maternal blood Ba levels following adjustment for covariates (*p* < 0.05, Table S2). Of these, 15 metabolites were positively correlated with Ba, including steroid metabolites (e.g., 7α,12α-dihydroxy-cholestene-3-one), aldehydes (e.g., cuminaldehyde), alcohols/alkaloids (e.g., hydrocotarnine), Diacylglycerols (DG(18:0/18:3)), fatty acid derivatives (e.g., 4-hydroxynonenal, dihomo-α-linolenic acid), and nucleotide-related metabolites (e.g., 5′-guanylic acid). Conversely, 34 metabolites were negatively correlated with Ba, encompassing key phospholipids such as Phosphatidylcholine (PC), Phosphatidylserine (PS), Phosphatidylethanolamine derivatives (PE-NMe/PE-NMe2), Sphingomyelins (SM), and Lysophosphatidylcholine (LysoPC), as well as several amino acid derivatives (e.g., N-acetyl-L-methionine, methyldopa), organic acids (e.g., isocitric acid, sebacic acid), and prostaglandins (e.g., Prostaglandin I2). Overall, significant associations emerged between Ba exposure and the decidual tissue metabolome. The 15 positively correlated metabolites were primarily linked to oxidative stress and lipid peroxidation, whereas the 34 negatively correlated metabolites were mainly associated with phospholipid and energy metabolism (Figure 2C).

In the MWAS analysis, after adjusting for all covariates, a total of 267 metabolites were found to be significantly associated with the risk of miscarriage (*p* < 0.05). Of these, 134 metabolites were positively correlated, while 133 metabolites were negatively correlated. These significantly associated metabolites were primarily distributed across 12 major metabolic categories, including amino acids, lipids, organic acids, and nucleotides. Specifically, the metabolite Deltorphin II showed the strongest association, with each 1 log10 increase in its concentration significantly increasing the risk of miscarriage (β = 0.13, 95% CI: 0.08–0.18). In contrast, the metabolite Methyltrienolone exhibited the most protective association (β = −0.15, 95% CI: −0.24–0.05). These significantly associated metabolites, along with their effect estimates and adjusted *p* values, are listed in Table S3, and the overall results are visualized in a volcano plot (Figure 2D). Figure 2G shows the heatmap of differential metabolites between the abortion group and the control group.

**Figure 3:**
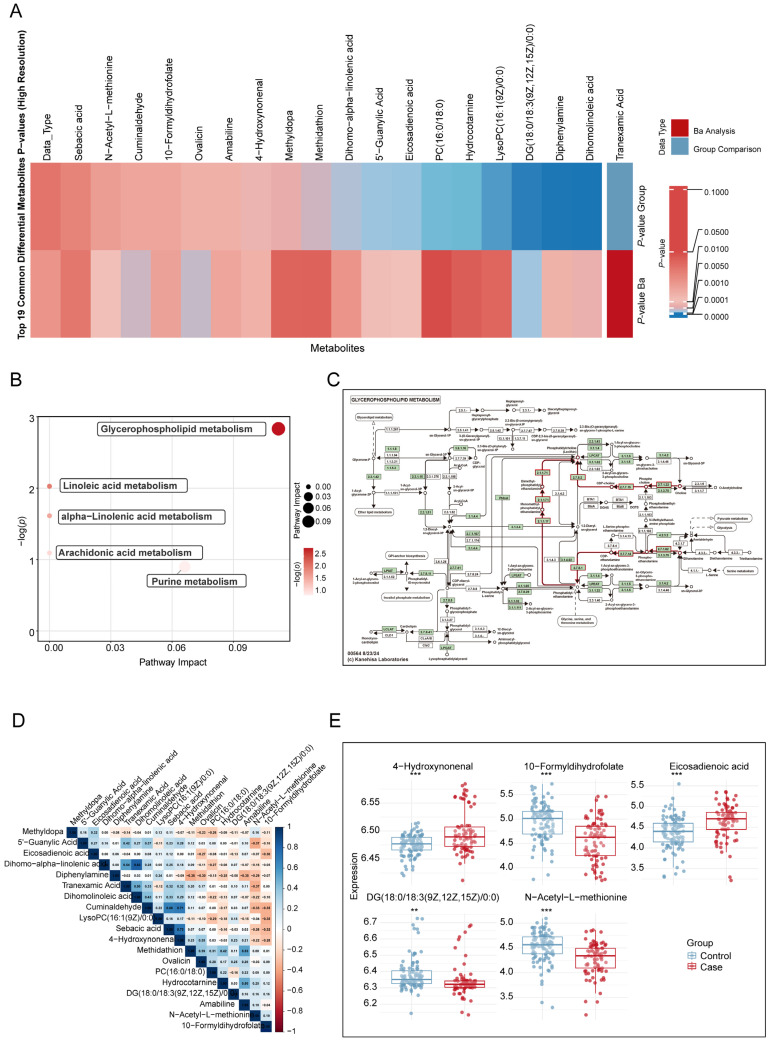
Overlapping metabolites in decidual tissue significantly associated with maternal barium exposure and miscarriage. (**A**). Heatmap analysis comparing the 19 overlapping metabolites identified in the MWAS of maternal blood Ba exposure and miscarriage using the MITM approach (**B**). KEGG analysis of metabolic pathways associated with maternal Ba exposure and miscarriage. (**C**). KEGG metabolic network map of glycerophospholipid metabolism. In this KEGG network map, the red lines indicate the glycerophospholipid metabolism, while other arrows and dotted lines follow standard KEGG notation. (**D**). Correlation matrix of 19 differential metabolites, calculated using *Spearman* correlation coefficients. (**E**). Differences in the concentrations of five signature metabolites between the miscarriage and control groups. Continuous variables were compared between groups using independent-samples *t*-tests after verifying normality assumptions. Covariates include maternal age, gestational age, pre-pregnancy BMI, education levels, and prior live births. MWAS: Metabolome-wide association study; MITM: Mediation Interaction Model. ** *p* < 0.01, *** *p* < 0.001.

Based on the MITM approach, 19 overlapping metabolites were identified between the maternal blood Ba exposure and miscarriage MWAS results. The overlapping metabolites included five amino acids, two bile acids, four ceramides, one fatty acid, one heterocycle, two lipids, one nucleotide, two phospholipids, one sphingolipid, and one vitamin (*p* < 0.05 and *P*_FDR_ < 0.2, Figure 3A and Table S4).

### 3.3. Overlapping Enriched Pathways Associated with Maternal Barium Exposure and Miscarriage

**Figure 4:**
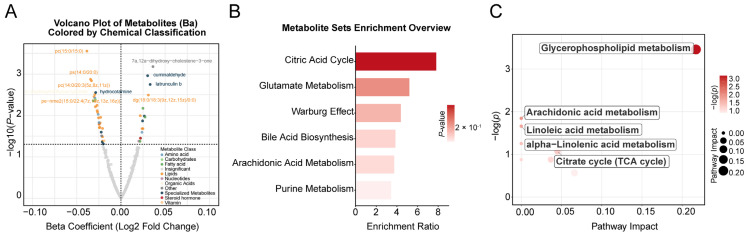
Metabolome-wide association study (MWAS) on barium and miscarriage. (**A**). A volcano plot showing the association between maternal barium exposure and metabolites in trophoblast tissue, analyzed by linear regression models. The *x*-axis represents the estimated coefficient of the association between maternal barium exposure and metabolites in trophoblast tissue, and the *y*-axis shows its -log10 (*p* value). The horizontal dotted line represents the significance threshold (*p* value = 0.05). (**B**). Overview of enriched metabolic pathways associated with barium exposure. (**C**). KEGG pathways significantly associated with barium exposure. Covariates include maternal age, gestational age, pre-pregnancy BMI, education levels, and prior live births.

Among the nine Kyoto Encyclopedia of Genes and Genomes (KEGG) pathways mapped, the glycerophospholipid metabolism pathway was significantly associated with maternal blood barium concentration (*p* < 0.05, Figure 4C and Table S5). Additionally, six metabolic pathways were significantly associated with miscarriage (*p* < 0.05, Figure 2E,F and Table S6): alanine, aspartate, and glutamate metabolism; phenylalanine metabolism; phenylalanine, tyrosine, and tryptophan biosynthesis; glyoxylate and dicarboxylate metabolism; glycerophospholipid metabolism; and arginine biosynthesis.

Based on the MITM approach, five metabolic pathways were identified as being enriched and simultaneously associated with both maternal barium exposure and miscarriage (Figure 3B and Table S7): glycerophospholipid metabolism (Figure 3C), linoleic acid metabolism, alpha-linolenic acid metabolism, arachidonic acid metabolism, and purine metabolism.

### 3.4. Mediating Role of Overlapping Metabolites in the Association Between Maternal Blood Ba Exposure and Miscarriage

To assess the potential mechanisms through which these overlapping metabolites mediate the association between maternal Ba exposure and miscarriage, we performed a mediation analysis. The results revealed that five metabolites exhibited significant mediating effects on the relationship between maternal Ba exposure and miscarriage (*p* < 0.05). These significant mediators were 5′-guanylic acid, cuminaldehyde, sebacic acid, 4-hydroxynonenal, and N-acetyl-L-methionine. The proportion of the total effect mediated by these metabolites ranged from 21.12% to 54.74%, with cuminaldehyde showing the highest share of the total mediating effect (Table 2 and Table S8).

### 3.5. Potential Biomarkers for Risk of Miscarriage

First, utilizing the LASSO logistic regression model, we determined the optimal λ value through five-fold cross-validation, which retained 16 variables with non-zero coefficients. Second, the RF algorithm was used, based on Gini Importance scores, to initially screen for the most significant metabolites. Subsequently, we integrated the results from both selection methods and performed a correlation analysis of the selected features to eliminate collinear variables with correlation coefficients (r) greater than 0.8 (Figure 3D and Table S9). Finally, by taking the intersection of the LASSO and RF results, we identified five core metabolites, N-acetyl-L-methionine, 4-hydroxynonenal, DG(18:0/18:3(9Z,12Z,15Z)/0:0), 10-formyldihydrofolate, and eicosadienoic acid, which served as the final feature set for predictive model construction (Figure 3E and Table S10).

**Figure 5:**
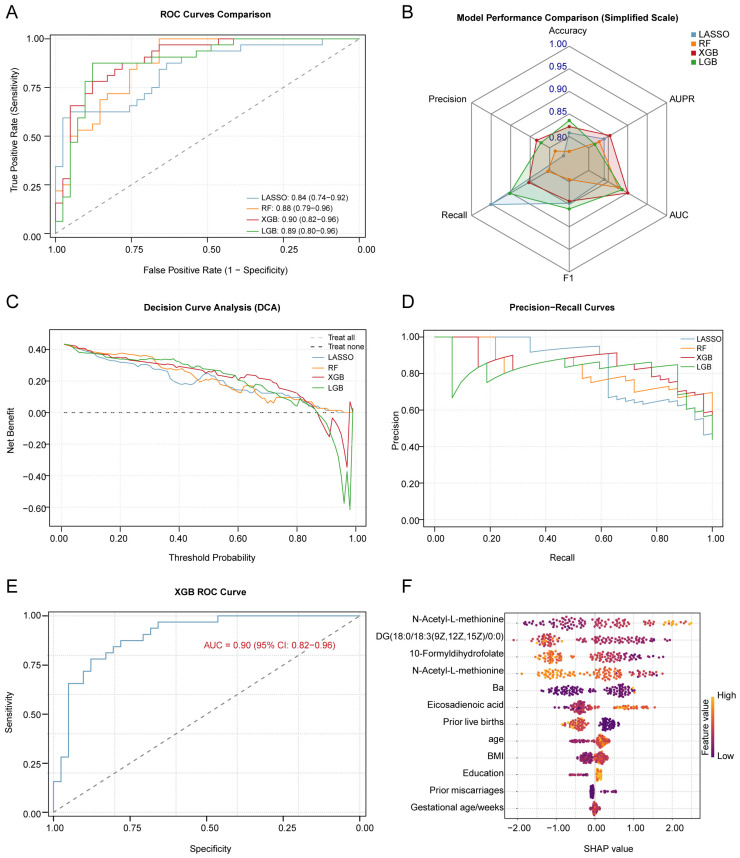
Performance evaluation of various machine learning models and detailed analysis of the best model. (**A**). Comparison of ROC curves for LASSO, RF, XGB, and LGB models, showing the trade-off between sensitivity and specificity. The *x*-axis represents the false positive rate (1-specificity), and the *y*-axis represents the true positive rate (sensitivity). (**B**). Comparison of LASSO, RF, XGB, and LGB models’ performance metrics, including accuracy, sensitivity, specificity, and AUC values (mean ± SD). Models were trained using 5-fold cross-validation. (**C**). DCA for LASSO, RF, XGB, and LGB models, showing the net benefit at various threshold probabilities. (**D**). Precision–Recall curves for LASSO, RF, XGB, and LGB models, evaluating model performance for imbalanced classes. (**E**). ROC curve of the best model, XGB, showing its accuracy in predicting the risk of miscarriage. (**F**). SHAP value peak plot for the best model, XGB, indicating the contribution of each feature to the prediction. The higher the SHAP value, the greater the impact of the feature on predicting the risk of miscarriage. ROC: Receiver Operating Characteristic Curve; AUC: area under curve; DCA: decision curve analysis; LASSO: Least Absolute Shrinkage and Selection Operator; RF: random forest; XGB, eXtreme Gradient Boosting; and LGB, Light Gradient Boosting Machine.

We adopted a stratified sampling method to split the data into training and testing sets at a 6:4 ratio. Four machine learning algorithms, LR, RF, XGBoost, and LightGBM, were used to build predictive models, with miscarriage as the dependent variable and the concentrations of Ba and the five core metabolites as the independent variables, further adjusting for covariates (Figure 5A and Table S11). Through five-fold cross-validation and evaluation on the testing set, we compared the performance metrics of the different models, including accuracy, recall, precision, and F1 score (Figure 5B and Table S12). Ultimately, the XGBoost model was selected as the final predictive model due to its superior performance across all metrics. On the testing set, the best-performing model (the XGBoost model) achieved an AUC of 0.90 (95% CI: 0.83–0.96) (Figure 5E, Figure S1 and Table S11). Calibration curve analysis demonstrated that the model’s predicted probabilities were highly consistent with the actual incidence rates (Figure S2). The learning curve indicated that the model’s performance plateaued with increasing sample size (Figure S2), suggesting no signs of significant underfitting or overfitting. Furthermore, the uniform and trendless distribution of the residual plot further confirmed the model’s goodness-of-fit. We assessed the model’s ability to predict positive cases by plotting the PR curve and calculating its AUPR, which reached 0.85 (Figure 5D). Finally, DCA results indicated that the model possessed high clinical utility (Figure 5C and Table S13).

## 4. Discussion

To our knowledge, this study was the first to systematically reveal the metabolic associations between maternal Ba exposure and miscarriage, providing new biological evidence for the impact of environmental pollutants on pregnancy outcomes. Building on our previous findings, which showed a significant positive association between the concentration of Ba in maternal whole blood and the risk of miscarriage, we further identified 19 metabolites that were significantly correlated with both Ba exposure and miscarriage. These metabolites mainly involve key biological processes such as oxidative stress, lipid peroxidation, and phospholipid metabolism. We also identified five metabolic pathways, with glycerophospholipid metabolism being the most prominent. Further results indicated that 5′-guanylic acid, cuminaldehyde, sebacic acid, 4-hydroxynonenal, and N-acetyl-L-methionine played significant mediating roles in the relationship between maternal Ba exposure and pregnancy loss. Finally, by utilizing metabolomics data, we identified five core metabolites, N-acetyl-L-methionine, 4-hydroxynonenal, DG(18:0/18:3(9Z,12Z,15Z)/0:0), 10-formyldihydrofolate, and eicosadienoic acid, which we used to construct a machine learning-based prediction model for pregnancy loss. The XGBoost model demonstrated excellent predictive performance on the test set (AUC = 0.90), suggesting its potential application value in clinical risk assessment.

We first investigated the metabolic characteristics of spontaneous abortion by comparing the trophoblast tissue metabolite profiles between the spontaneous abortion group and the healthy pregnancy group. The results of metabolomic differential analysis indicated significant abnormalities in several key metabolic pathways in the spontaneous abortion group, with a notable enrichment in amino acid metabolism and glycerophospholipid metabolism. During pregnancy, the demand for amino acids increases, and their proper functioning and metabolic pathways are crucial for maintaining maternal health and supporting normal fetal development [29]. Specifically, the aberrant metabolism of tyrosine may affect thyroid function, which in turn could indirectly influence maternal health and increase the risk of adverse pregnancy outcomes [29,30]. In the placenta, tryptophan is metabolized through the serotonin and kynurenine pathways, generating metabolites with neuroactive, immunosuppressive, vasculature-modulating, and redox properties [30,31]. The biosynthesis and metabolism of arginine are critical for embryo vitality, fetal growth, and immune function [32,33,34,35]. Furthermore, glutamine metabolism is vital for a functional maternal–fetal interface in early pregnancy, and its disruption increases miscarriage risk [36]. Studies have reported significantly reduced levels of glutamine metabolism in the endometrium of women with recurrent spontaneous abortion, a phenomenon that was also confirmed in our metabolomic analysis [37,38]. Our findings on lipid metabolism align with existing lipidomics data, indicating a close association between glycerophospholipid metabolism dysregulation and adverse pregnancy outcomes. These results further support the crucial role of metabolic imbalance in the pathogenesis of spontaneous abortion and provide potential biological evidence for related intervention measures [39,40,41].

**Figure 6:**
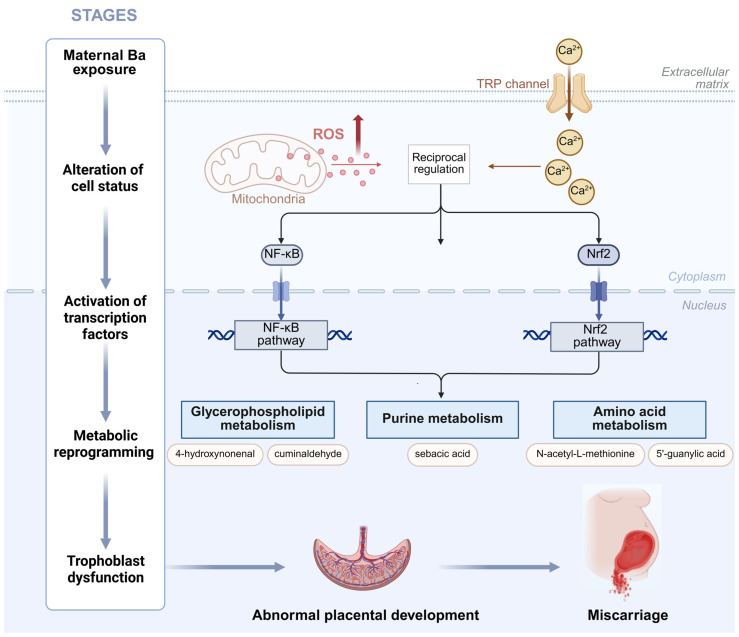
A model hypothesis diagram demonstrating the mechanism of Ba exposure leading to miscarriage.

Our findings align with a substantial body of literature documenting the role of environmental pollutants in disrupting pregnancy maintenance. Epidemiological and toxicological studies have shown that heavy metal pollutants primarily induce oxidative stress and disrupt lipid metabolism, leading to organ dysfunction [13,14,42]. Ba has been reported to cross the placental barrier, directly impacting fetal development, inducing oxidative stress by increasing glutathione reductase activity and malondialdehyde (MDA) levels [13,43,44]. Emerging evidence further suggests that Ba-induced oxidative stress may trigger a more complex cascade of molecular events. Lipid peroxidation, a key consequence of oxidative stress, generates reactive aldehydes such as 4-hydroxynonenal (4-HNE). These aldehydes not only act as damage products but also function as signaling molecules, forming adducts with key regulators such as Keap1 and IKK [45]. These interactions directly influence critical pathways such as Nrf2/ARE and NF-κB, creating a damaging feedforward loop that links oxidative damage to inflammation and metabolic dysregulation [46]. Moreover, sustained oxidative stress can lead to the depletion of glutathione (GSH), which in turn inhibits glutathione peroxidase 4 (GPX4), potentially sensitizing placental cells to ferroptosis [47]. The enriched metabolic pathways identified in our study included glycerophospholipid, linoleic acid, alpha-linolenic acid, and arachidonic acid metabolism, suggesting lipid peroxidation as a central event in Ba-induced toxicity. This oxidative damage compromised placental cell membrane integrity, injured the vascular endothelium, and caused DNA, lipid, and protein damage in placental tissues, ultimately contributing to embryonic arrest [14]. These processes led to premature placental aging, higher vascular resistance, inadequate blood perfusion, and fetal hypoxia, which predispose to spontaneous abortion and other adverse pregnancy outcomes [48,49]. Furthermore, altered purine metabolism pathways indicated disturbances in nucleic acid synthesis and damage repair, critical for early embryogenesis and fetal development [43,44]. Additionally, Ba’s potential estrogen-like effects [50] could synergize with oxidative stress and metabolic disturbances to promote miscarriage. Integrating the available evidence, we propose a refined molecular hypothesis: Ba exposure is likely to initiate toxicity by disrupting cellular ion homeostasis, which subsequently leads to mitochondrial dysfunction and the excessive generation of Reactive Oxygen Species (ROS). This oxidative stress activates key pathways, including Nrf2/ARE and NF-κB, which in turn drive metabolic reprogramming in lipid, purine, and one-carbon metabolism. These disturbances impair trophoblast function, hinder placental development, and compromise embryo viability, ultimately culminating in miscarriage (Figure 6).

Mediation analysis further highlighted the key roles of 5′-guanylic acid, cuminaldehyde, sebacic acid, 4-hydroxynonenal, and N-acetyl-L-methionine in the association between Ba exposure and spontaneous abortion. Among these, 4-hydroxynonenal (4-HNE), a hallmark product of lipid peroxidation, has been repeatedly shown in previous studies to be significantly associated with adverse pregnancy outcomes, such as recurrent miscarriage, preeclampsia, and placental insufficiency [49,51,52]. Elevated levels of 4-HNE serve as a clear signal of severe oxidative damage to cells. Abnormal levels of sebacic acid, a product of fatty acid oxidation, indicate impaired lipid catabolic pathways, and lipid metabolism dysfunction is closely linked to embryo implantation failure and placental developmental abnormalities [53]. Disturbances in 5′-guanylic acid (GMP) point to purine-pathway dysregulation [54]. The developing placenta or embryo relies on high, dynamically regulated de novo purine synthesis for nucleic acid biosynthesis and trophoblast differentiation [55], so purine deficiencies can constrain proliferation programs essential for normal development. Changes in N-acetyl-L-methionine levels point to impaired antioxidant defense and methylation capacity, contributing to disruptions in one-carbon metabolism, which is related to DNA synthesis and methylation. Defects in one-carbon metabolism are a significant risk factor for spontaneous abortion [56,57,58]. Cuminaldehyde may reflect the active state or load of liver detoxification systems (such as aldehyde oxidase and alcohol dehydrogenase) under the high oxidative stress induced by Ba exposure [43,44,59]. Ba exposure may affect liver function, weakening the body’s ability to metabolize and eliminate such exogenous compounds, and this impaired detoxification capacity, or reduced metabolic flexibility, could synergize with the mechanisms underlying adverse pregnancy outcomes. These mediating metabolites together form a network of multi-pathway disruptions, serving as the core link connecting environmental exposure to disease development.

Although the predictive accuracy of the metabolomics model was slightly lower compared to chemical exposure-based models, it demonstrated irreplaceable importance in elucidating potential disease mechanisms and identifying biomarkers. In this study, four machine learning algorithms were utilized to construct a spontaneous abortion risk prediction model based on five core metabolites (N-acetyl-L-methionine, 4-hydroxynonenal, DG(18:0/18:3(9Z,12Z,15Z)/0:0), 10-formyldihydrofolate, eicosadienoic acid), and the best-performing model, XGBoost, was ultimately selected. The combined biomarker panel consisting of these five metabolites strongly supported and validated the core mechanistic hypotheses of this study. DG(18:0/18:3(9Z,12Z,15Z)/0:0) and eicosadienoic acid reflect an imbalance in membrane lipid composition, with their changes associated with inflammation and compromised membrane integrity. 10-Formyldihydrofolate can serve as an alternative donor for methionyl-tRNA formyltransferase, thereby reshaping one-carbon/folate metabolism and modulating antifolate responses, while defects in 10-formyltetrahydrofolate dehydrogenase may impair folate handling and are associated with neonatal hydrocephalus [60,61].

This model demonstrates significant value for clinical translation. By integrating multiple metabolite information, it enables earlier and more precise miscarriage risk assessment compared to single clinical indicators, thus providing strong support for the early identification and precise stratification of women with high-risk pregnancies. This study systematically revealed the potential relationship between maternal Ba exposure and spontaneous abortion through an MWAS. Unlike conventional environmental exposure studies, the innovative aspect of this work lies in utilizing metabolomic profiling from whole-blood samples to explore the underlying mechanisms through which Ba exposure influences spontaneous abortion via metabolic pathways. In constructing the predictive model, we integrated four machine learning algorithms—LR, RF, XGBoost, and LightGBM—and comprehensively evaluated their performance in predicting the risk of spontaneous abortion associated with Ba exposure through comparative analysis. Notably, this study also employed two complementary feature selection strategies, LASSO logistic regression and random forest, which effectively identified five core metabolites as the final feature set. This approach fully accounted for interrelationships among metabolites, thereby avoiding potential biases inherent in single-method strategies.

Although this study provided significant findings, its limitations must be carefully considered. First, this study employed a cross-sectional design. Although a significant association between barium exposure and spontaneous abortion was observed, such an observational design precludes causal inference. To further elucidate the causal role of barium exposure, future research should adopt more rigorous longitudinal designs or combine animal model experiments to validate its causal effects on pregnancy loss. The risk prediction model constructed in this study is essentially a retrospective model with high discriminative power based on post-outcome data. Whether the identified metabolic markers exhibit abnormalities prior to miscarriage and possess genuine early-warning value remains to be verified in future, independently designed prospective cohort studies, particularly by collecting biospecimens in early pregnancy, well before the occurrence of miscarriage. Second, the sample size in this study was relatively small, which may limit the generalizability of the results. Additionally, this study did not collect detailed information on several potential confounders, including participants’ habitual physical activity levels and specific histories of occupational exposure to environmental pollutants. Although occupational records indicated that the majority of participants were employed in non-industrial professions, the absence of quantitative or individualized exposure assessments prevented a comprehensive adjustment for these factors in our analysis. Future studies should address these limitations by more thoroughly considering these variables. The concentration of Ba in whole blood was used as the primary exposure indicator, which, while reflective of maternal Ba exposure levels, does not fully represent placental or fetal exposure. Future studies could utilize placental or umbilical cord blood samples in supplementary exposure assessments to further validate Ba exposure concentrations in different biological samples and their impact on miscarriage. In the real-world environment, Ba often co-occurs with other environmental pollutants (e.g., PM_2.5,_ organic pollutants), meaning that co-exposure to multiple agents is likely. The interaction of these pollutants may influence miscarriage risk, but such complex environmental factors were not considered in this study. Future research could comprehensively consider the exposure to multiple pollutants and metabolomic changes to further uncover the combined impact of multiple exposures on spontaneous abortion. This study primarily focused on the metabolic mechanisms of maternal barium exposure. Increasing evidence from metabolomics studies suggests that environmental pollutants can reshape the metabolic profiles of the testes and directly damage sperm chromatin [4,62]. Future research could provide a more comprehensive understanding of the complex relationship between environmental exposure and reproductive health by integrating assessments of paternal environmental exposure and sperm quality.

## 5. Conclusions

This study demonstrated that maternal Ba exposure was associated with widespread disruptions in decidual tissue metabolism, particularly in glycerophospholipid and amino acid pathways, which were linked to an increased risk of miscarriage. Key metabolites involved in oxidative stress, lipid peroxidation, and one-carbon metabolism mediate this relationship. Integrating metabolomics data with machine learning served as a powerful approach for identifying biomarker panels and elucidating potential mechanisms underlying Ba-induced pregnancy loss. These findings enhance our understanding of how environmental exposures contribute to miscarriage and provide a foundation for future research into early risk detection and preventive strategies.

## Figures and Tables

**Table 1:** Characteristics of pregnant women in the study.

Characteristics	N (%)/Mean ± SD		*p* Value
	Control *N* = 101	Miscarriage *N* = 82	
Age (years)	31.03 (5.76)	31.38 (5.25)	0.673
Pre-pregnancy BMI (kg/m^2^)	21.58 (2.97)	21.70 (2.39)	0.778
Gestational age (weeks)	8.62 (1.09)	9.00 (1.15)	0.025
Prior pregnancies			0.106
0	23 (22.8)	31 (37.8)	
1	24 (23.8)	17 (20.7)	
2	27 (26.7)	13 (15.9)	
≥3	27 (26.7)	21 (25.6)	
Prior miscarriages			0.003
0	92 (91.1)	59 (72.0)	
1	6 (5.9)	18 (22.0)	
≥2	3 (3.0)	5 (6.1)	
Prior live births			
0	33 (32.7)	51 (62.2)	<0.001
1	52 (51.5)	29 (35.4)	
≥2	16 (15.8)	2 (2.4)	
Education (%)			
Lower than high school	3 (3.0)	6 (7.3)	0.1
High school/some college	21 (20.8)	9 (11.0)	
College graduate	21 (20.8)	25 (30.5)	
Graduate degree or higher	56 (55.4)	42 (51.2)	
Occupation			0.618
Office work	55 (54.5)	48 (58.5)	
Services/Workers	2 (2.0)	4 (4.9)	
Professionals	4 (4.0)	5 (6.1)	
Unemployed	22 (21.8)	17 (20.7)	
Students	4 (4.0)	1 (1.2)	
Others	14 (13.9)	7 (8.5)	
Personal income (CNY)			0.705
<1000	27 (26.7)	20 (24.4)	
1000–4999	25 (24.8)	18 (21.9)	
5000–9999	37 (36.6)	28 (34.1)	
≥10,000	12 (11.9)	16 (19.5)	
Smoking during pregnancy			0.240
Yes	2 (1.9)	1 (1.2)	
No	99 (98.0)	81 (98.8)	
Alcohol consumption during pregnancy			0.285
Yes	5 (5.0)	2 (2.4)	
No	96 (95.0)	80 (97.6)	

**Note:** SD, standard deviation; continuous variables are presented as the means ± SD; categorical variables are presented as proportions [N (%)]; BMI, body mass index; CNY, Chinese Yuan; *p* values were derived from the *t*-test.

**Table 2:** Analyses of the meditating role of blood metabolites in the association between maternal Ba exposure and an increased risk of miscarriage.

Mediator	Indirect Effect β (95% CI)	*p* Value	Proportion Mediated
5′-Guanylic acid	0.003 (0.001–0.008)	<0.0001	21.1%
Cuminaldehyde	0.007 (0.003–0.011)	<0.001	54.7%
Sebacic acid	0.007 (0.002–0.012)	<0.005	48.3%
4-Hydroxynonenal	0.005 (0.002–0.009)	<0.005	33.0%
N-Acetyl-L-methionine	0.005 (0.001–0.008)	<0.005	29.5%

**Note:** The model was adjusted for maternal age, gestational age, pre-pregnancy BMI, education level, and parity. CI, confidence interval.

## Data Availability

The data that support the findings will be available in [OMIX, China National Center for Bioinformation/Beijing Institute of Genomics, Chinese Academy of Sciences] at [https://ngdc.cncb.ac.cn/omix: accession no.OMIX012565] following an embargo from the date of publication to allow for commercialization of research findings.

## Supplementary Materials

The following supporting information can be downloaded at https://www.mdpi.com/article/10.3390/toxics13121066/s1, Figure S1. Performance evaluation and feature interpretation of the risk of miscarriage prediction model based on the XGBoost algorithm. Figure S2. Comparative performance and comprehensive evaluation of the risk of miscarriage prediction models based on multiple machine learning algorithms. Table S1. Chemical Annotation and Classification Database. Table S2. Complete Metabolome-Wide Association Study Results for Barium Exposure. Table S3. Integrated Metabolome-Wide Association Study Results Across Comparative Groups. Table S4. Barium-Associated Metabolites with Chemical Category Annotations. Table S5. KEGG Pathway Enrichment Analysis for Barium-Associated Metabolites. Table S6. KEGG Pathway Analysis for Miscarriage Metabolites. Table S7. Shared KEGG Pathways Between Barium Exposure and Miscarriage. Table S8. Mediation Analysis of Barium Exposure on Miscarriage. Table S9. Comprehensive Metabolite-Metabolite Correlation Matrix. Table S10. Random Forest Feature Importance Rankings for Metabolite Predictors. Table S11. ROC Area Under Curve Values with Confidence Intervals. Table S12. Comparative Performance Metrics of Multiple Prediction Models. Table S13. Decision Curve Analysis Data for Clinical Utility Assessment.
