# Supplementary material for: Metabolomic Impact of Maternal Barium Exposure on Miscarriage Risk: Identification of Metabolic Biomarkers and Construction of a Risk Prediction Model
Source: Toxics. 2025 Dec 10;13(12):1066. doi: 10.3390/toxics13121066 (PMC12737389; doi:10.3390/toxics13121066)
Supplement: Supplementary file 1 [file toxics-13-01066-s001.zip › toxics-4003465-supplementary figures.pdf]

Supplementary materials

Metabolomic impact of maternal barium exposure on miscarriage: Identification of metabolic biomarkers and construction of a risk prediction model

Xiaoyu Zhao [a], Ziwei Guo [a], Shuangshuang Zhao [a], Danyang Wan [b, c], Jie Xu [a], Yifan Xu [b, c], Yujie Liu [b, c], Haoyi Xu [a], Ziyang Wang [a], Qing Xu [a, *]

Contact the corresponding author: Qing Xu, xuqing@njmu.edu.cn

Table of contents

Figure S1. Performance evaluation and feature interpretation of the risk of miscarriage prediction model based on the XGBoost algorithm.

Figure S2. Comparative performance and comprehensive evaluation of the risk of miscarriage prediction models based on multiple machine learning algorithms.

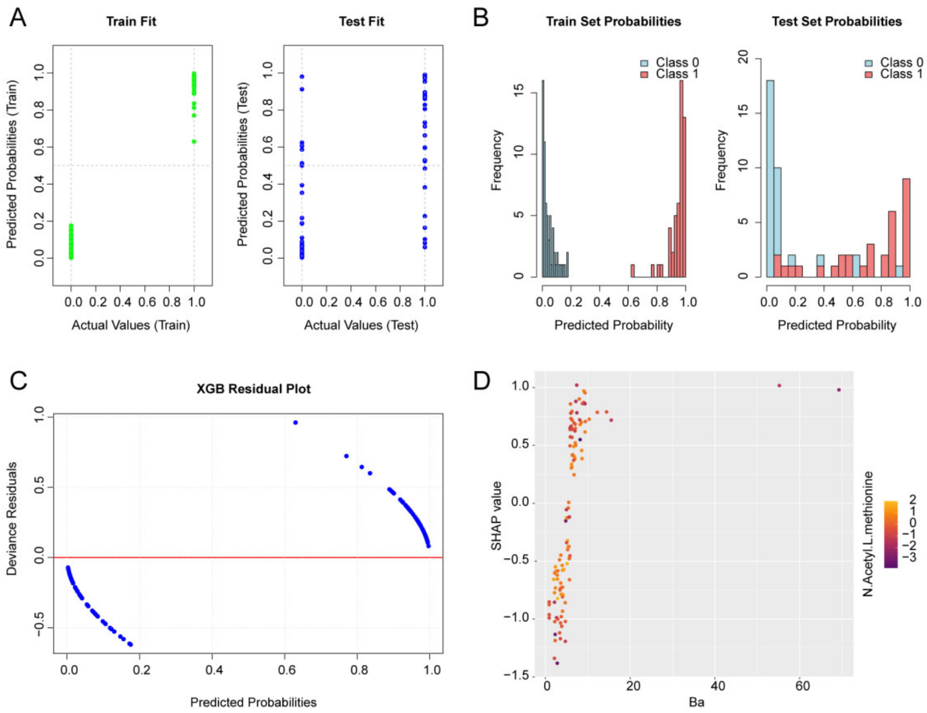

Figure S1. Performance evaluation and feature interpretation of the risk of miscarriage prediction model based on the XGBoost algorithm.

A. Predicted Probability Fit Plots for Training and Test Sets. Panel A illustrates the fit between the predicted probabilities and the actual outcome labels (0 or 1) in both the training and test sets. An ideal prediction would result in points clustered strictly at 0 and 1;

B. Distribution of Predicted Probabilities for Training and Test Sets. Panel B displays the frequency histograms of predicted probabilities for the case (Class 1) and control (Class 0) groups in both the training and test sets. Ideally, the probability distributions for the two classes should be clearly separated;

C. XGBoost Residual Plot. Panel C shows the relationship between the XGBoost model's predicted probabilities and the Deviance Residuals. This plot is used to evaluate the goodness-of-fit and the distribution pattern of the residuals;

D. SHAP Value Analysis of Core Feature Contribution and Exposure Level Association. Panel D illustrates the SHAP value contribution of Ba exposure and N-Acetyl-L-methionine (a core mediating metabolite) to the model's prediction. The color gradient (N-Acetyl-L-methionine levels) and the X-axis (Ba concentration)

collectively reveal how varying levels of the exposure and the metabolite influence the prediction outcome.

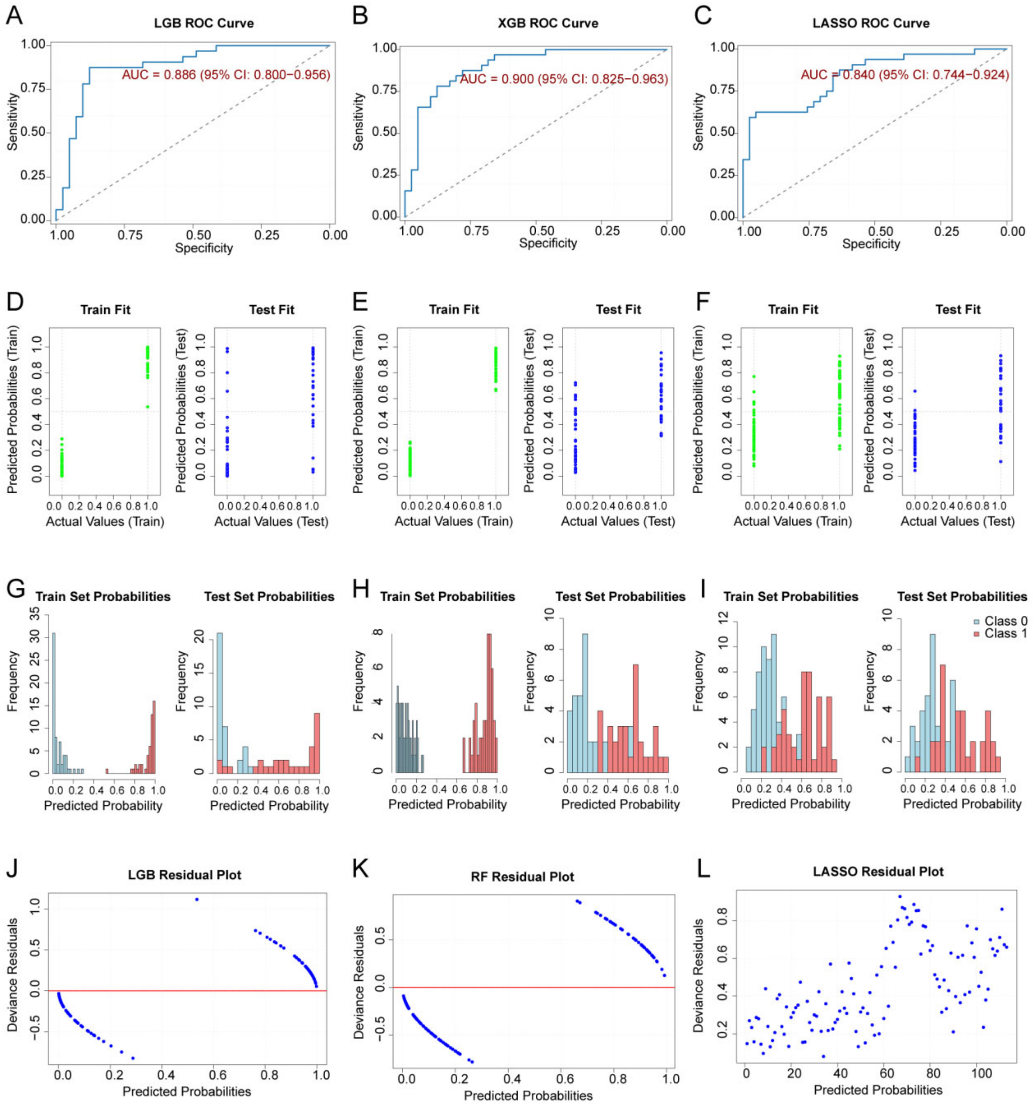

Figure S2. Comparative performance and comprehensive evaluation of the risk of miscarriage prediction models based on multiple machine learning algorithms.

A. LightGBM (LGB) Model ROC Curve: Displays the LGB model's True Positive Rate (Sensitivity) versus False Positive Rate (1−Specificity) across various classification thresholds. The plot indicates the model's AUC value and its 95% Confidence Interval (CI), used to assess the overall discriminative ability of the model.

B. XGBoost (XGB) Model ROC Curve: Displays the XGB model's ROC curve and AUC value (and its 95% CI), used to evaluate its ability to distinguish between

spontaneous abortion and normal pregnancy.

C. LASSO Logistic Regression Model ROC Curve: Displays the LASSO model's ROC curve and AUC value (and its 95% CI) on the dimensionally reduced feature set.

D. LGB Model Predicted Probability Fit Plot: Shows the distribution of predicted probabilities versus actual outcome labels (0 or 1) for the LGB model on the training set (green dots) and the test set (blue dots). Better fit is indicated by points clustered closer to the actual label values (0 or 1).

E. XGB Model Predicted Probability Fit Plot: Shows the predicted probability fit for the XGB model on the training and test sets, used to evaluate the model's fitting effect and generalization capability.

F. LASSO Model Predicted Probability Fit Plot: Shows the predicted probability fit for the LASSO model on the training and test sets.

G. LGB Model Predicted Probability Density Distribution Plot: Displays, using histograms, the distribution of LGB model predicted probabilities for the control group (Class 0, blue) and the case group (Class 1, red). Higher separation between the two distributions indicates better model discrimination.

H. XGB Model Predicted Probability Density Distribution Plot: Displays the distribution of XGB model predicted probabilities for the control group and the case group.

I. LASSO Model Predicted Probability Density Distribution Plot: Displays the distribution of LASSO model predicted probabilities for the control group and the case group.

J. LGB Model Residual Plot: Shows the relationship between the LGB model's predicted probabilities and the Deviance Residuals. This plot is used to assess the goodness-of-fit of the model.

K. Random Forest (RF) Model Residual Plot: Shows the relationship between the RF model's predicted probabilities and the Deviance Residuals, used to assess the fit quality of the RF model.

L. LASSO Model Residual Plot: Shows the relationship between the LASSO model's predicted values and the Deviance Residuals. Ideally, residuals should be randomly

distributed around 0 with no clear pattern.
